# Effects of a low-sodium diet in patients with idiopathic hyperaldosteronism: a randomized controlled trial

**DOI:** 10.3389/fendo.2023.1124479

**Published:** 2023-04-19

**Authors:** Lihua Zhou, Yiran Jiang, Cui Zhang, Tingwei Su, Lei Jiang, Weiwei Zhou, Xu Zhong, Luming Wu, Weiqing Wang

**Affiliations:** ^1^ Shanghai Key Laboratory for Endocrine Tumors, Shanghai Clinical Center for Endocrine and Metabolic Diseases, Key Laboratory for Endocrine and Metabolic Diseases of the Chinese Health Ministry, Ruijin Hospital, Shanghai Jiao Tong University School of Medicine, Shanghai, China; ^2^ Laboratory for Endocrine and Metabolic Diseases, Institute of Health Sciences, Shanghai Jiao Tong University School of Medicine, and Shanghai Institutes for Biological Sciences, Chinese Academy of Sciences, Shanghai, China

**Keywords:** idiopathic hyperaldosteronism, low sodium diet, sodium, blood pressure, potassium, randomized controlled trial

## Abstract

**Background:**

Idiopathic hyperaldosteronism (IHA) is one of the most common types of primary aldosteronism (PA), an important cause of hypertension. Although high dietary sodium is a major risk factor for hypertension, there is no consensus on the recommended dietary sodium intake for IHA.

**Objective:**

This study investigated the effect of a low-sodium diet on hemodynamic variables and relevant disease biomarkers in IHA patients, with the aim of providing a useful reference for clinical treatment.

**Methods:**

Fifty IHA patients were evenly randomized into two groups and provided, after a 7-day run-in period (100 mmol/d sodium), either a low-sodium diet (50 mmol/d sodium) or a normal sodium diet (100 mmol/d sodium) for an additional 7 days. After the 14-day intervention (conducted without potassium supplementation), changes in blood pressure (BP) and serum potassium were evaluated in both groups.

**Results:**

After the dietary intervention, the low sodium group exhibited, compared to the normal sodium group, decreased BP (SBP: 121.8 ± 12.8 *vs*. 129.9 ± 12.1 mmHg, p < 0.05; DBP: 82.6 ± 7.6 *vs*. 86.4 ± 8.2 mmHg, p < 0.05; MAP: 95.7 ± 8.8 *vs*. 100.9 ± 8.4 mmHg, p < 0.05) and increased serum potassium levels (3.38 ± 0.33 *vs*. 3.07 ± 0.27 mmol/L, p < 0.001). The low sodium group showed also better control of both BP and serum potassium: BP <140/90 mmHg in 70.0% of total patients (76.0% *vs*. 64.0%, in the low and normal sodium groups, respectively; p > 0.05), BP <130/85 mmHg in 38.0% of total patients (56.0% *vs*. 20.0%, p < 0.05), BP <120/80 mmHg in 28.0% of total patients (44.0% *vs*. 12.0%, p < 0.05); serum potassium ≥3.5 mmol/L in 22.0% of total patients (32.0% *vs*. 12.0% in the low and normal sodium groups, respectively; p = 0.088). There were differences between the controlled BP group (<120/80 mmHg) and the non-controlled BP group (≥120/80 mmHg) in gender, BP at baseline, and type of diet (low *vs*. normal sodium). Female gender and low-sodium diet were protective factors for BP control.

**Conclusions:**

A low-sodium diet is effective in lowering BP and elevating serum potassium in IHA patients. Female patients on a low-sodium diet are more likely to achieve BP control (<120/80 mmHg). We advocate a dietary sodium intake of 50 mmol/d for IHA patients.

**Clinical trial registration:**

https://clinicaltrials.gov, Identifier NCT05649631.

## Introduction

Primary aldosteronism (PA) is considered the most common form of endocrine hypertension, accounting for 4%–10% of hypertension cases (1, 2). PA is characterized by hypertension, low serum potassium, and high plasma aldosterone concentration (PAC). PA patients are at greater risk of cardiovascular, metabolic, and renal disease than those with essential hypertension (3–8). Subtypes of PA include aldosterone-producing adenoma (APA), unilateral adrenal hyperplasia (UAH), bilateral adrenal hyperplasia (BAH; also called idiopathic hyperaldosteronism, IHA), adrenocortical carcinoma, and familial hyperaldosteronism. Among these PA subtypes, APA and IHA are the two most common forms (~90% of PA cases) (9). Unilateral PA (APA and UAH) is treated with surgery, while IHA can usually be treated by medication. Spironolactone, a competitive antagonist of the mineralocorticoid receptor and a potassium-preserving diuretic, is commonly used to treat PA. However, it has dose-dependent side effects, including gynecomastia and impotence in males and menstrual irregularities in females. Results from early studies showed that the incidence of gynecomastia was 10% at a spironolactone dose of 25 mg/day (10), and 52% or more at a dose of 150 mg/day (11). Thus, alternative or adjuvant therapies to reduce the dose of spironolactone are in urgent need.

Sodium in diet is mainly derived from NaCl (salt) and is essential for cellular homeostasis and body function. One gram of sodium is contained in ~2.5 g of salt, and almost 90% of sodium in the diet comes from table salt. Both the World Health Organization (WHO) (12) and the European Society of Hypertension/European Society of Cardiology (ESH/ESC) (13) recommend a daily salt intake of no more than 5 g (equivalent to 87 mmol/L of sodium) in the general population and in hypertensive patients. However, research has showed that in most countries the average salt intake is ~10 g/d (14, 15). Sodium intake is particularly high among Chinese people. In a large research study that included population samples from East Asian and Western countries, the Beijing population had the highest average sodium intake, reaching 300 mmol/d for males and 250 mmol/d for females (i.e. 17.6 and 14.8 g of salt, respectively) (16).

Aldosterone excess has a marked effect on taste perception. A prospective study found that NaCl taste perception was significantly impaired in patients with PA, which evidenced a NaCl taste recognition threshold about twice as high as that observed in essential hypertension (17). In a retrospective study, it was estimated that PA patients consumed at least 10 g of salt per day in their diet, with significantly higher mean intake reported for unilateral PA compared to bilateral PA (11.9 g/d *vs*. 10.4g/d, respectively) (18). There is a direct linear relationship between salt intake and risk of cardiovascular disease and/or death (19–21), and several studies have shown that sodium is an important causative factor in essential hypertension (22–25). Thus, target organ deterioration is greatly increased by both hyperaldosteronism and high sodium intake (26).

Reducing sodium intake in essential hypertensive patients has been shown to lower blood pressure (BP) (27) and reduce cardiovascular events (28). Similarly, reducing sodium intake in patients with PA can significantly improve left ventricular mass index (29). Regulation of sodium and potassium homeostasis occurs primarily in the renal tubules, and high sodium intake determines increased renal excretion of potassium (30, 31). Therefore, we conducted the present trial based on the hypothesis that in patients with IHA serum potassium would be increased by reducing sodium intake.

A reduction in sodium intake is usually followed by a drop in blood volume, which activates the renin-angiotensin-aldosterone system (RAAS) (32, 33). A study has shown a mild increase in plasma renin activity (PRA) after moderate reduction in sodium intake (34), but it is not clear whether a similar change may occur in PA. A retrospective study showed that elevated PRA contributed to a reduction in cardiovascular events (35), and similar findings were found in an observational study (36). Hence, in IHA patients an elevation in PRA after reducing sodium intake would represent a positive effect.

The current guidelines for PA do not specify a recommended intake of sodium, and the effect of reduced sodium intake on PA is unclear. Based on this, and the above considerations, the goal of this study was to investigate the effect of a low-sodium diet on IHA and to provide a useful reference for clinical practice.

## Methods

### Patients

**Figure 1:**
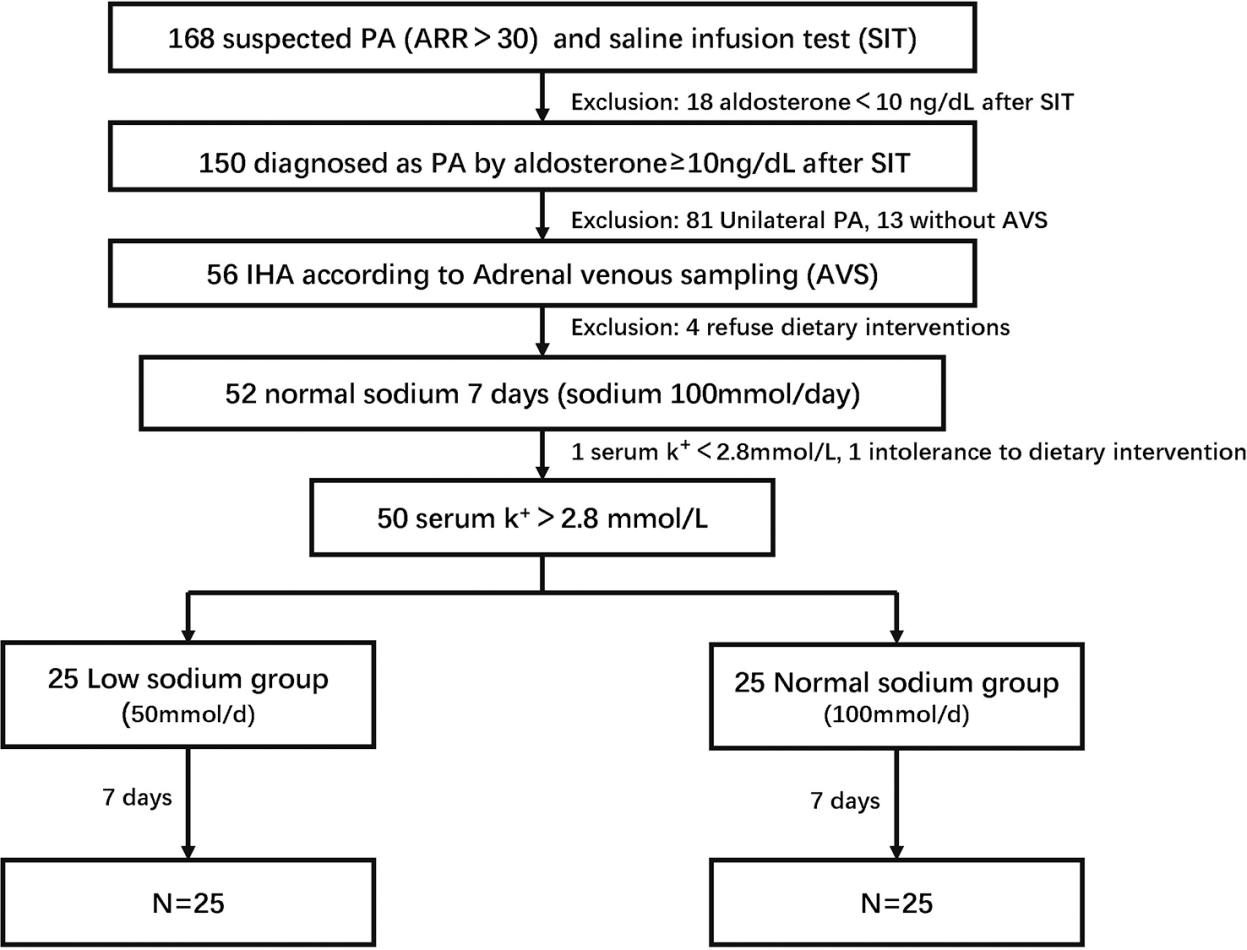
Flow chart of the study.

We screened 168 inpatients (all of them are Chinese Han population) with suspected PA who were admitted to Ruijin Hospital Affiliated to Shanghai Jiao Tong University School of Medicine from July 2021 to September 2022. A total of 150 patients diagnosed with PA by saline infusion test (SIT) were initially evaluated for study inclusion. According to adrenal computed tomography (CT) and adrenal venous sampling (AVS), we identified 81 UPA, 56 IHA, and 13 unclassifiable PA cases. None of the patients had restricted their sodium intake before definitive subtype diagnosis. We conducted a dietary intervention on 52/56 IHA cases, as four patients refused to participate in the trial. Two patients withdrew from the trial during its run-in period (one with serum potassium <2.8 mmol/L and another who could not tolerate the dietary arrangement). A total of 50 IHA patients were randomly assigned to either a low sodium group or a normal sodium diet group (25 per group) and completed the dietary intervention trial (**Figure 1**). This study was approved by the Ethics Committee of Ruijin Hospital, Shanghai Jiao Tong University of Medicine, and all patients provided written informed consent to participate.

### Enrollment criteria

Inclusion criteria were: 1) 18–70 years of age; 2) diagnosed as PA by SIT; 3) no lateralization of aldosterone secretion according to AVS; and 4) serum potassium ≥ 2.8 mmol/L after the run-in period. Exclusion criteria included: 1) impaired renal function [creatinine clearance rate (Ccr) <60 ml/min]; 2) impaired liver function [alanine transaminase (ALT) and aspartate transaminase (AST) >2.5 times upper limit of normal]; 3) patients with heart failure [New York Heart Association (NYHA) ≥class 3 or ejection fraction (EF) <50%]; 4) patients with stroke or acute infarction in the last 6 months; 5) patients who are pregnant or breastfeeding; 6) patients who cannot tolerate the dietary arrangements; and 7) patients with history of malignant tumors in the last 6 months.

### Diagnosis criteria

PA was diagnosed according to the 2016 Endocrine Society Clinical Practice Guidelines (9). Patients with aldosterone-to-renin ratio (ARR) >30 (ng/dl)/(mg/dl/h) underwent a confirmatory test (SIT). Diagnosis of PA was established if PAC >10 ng/dl after SIT. Adrenal CT and AVS were used to differentiate between unilateral PA and bilateral PA. Cannulation was considered successful if the cortisol _adrenal vein_/cortisol _peripheral vein_ ratio was greater than 3 without adrenal corticotropic hormone (ACTH) stimulation. The cortisol-corrected aldosterone (A/C) ratio served to correct adrenal venous aldosterone levels for differing degrees of dilution of adrenal *vs*. peripheral venous blood. (A/C) _adrenal vein_/(A/C) _contralateral adrenal vein >_2 was considered as evidence of dominant aldosterone secretion. IHA was diagnosed by: 1) a biochemical diagnosis of PA; and 2) no lateralization of aldosterone secretion according to AVS.

### Research design

This study was a single-center randomized controlled trial which lasted 14 days and consisted of two stages (run-in period and intervention period), each one comprising 7 days without potassium supplementation. If participants met the enrollment criteria at the end of the run-in period, they were randomly assigned (random number method) 1:1 to the low sodium group (50 mmol/d sodium) or the normal sodium group (100 mmol/d sodium) until the end of the intervention period. We took into account both the sodium contained in the food and the sodium in the salt. The primary outcome was the change in BP and serum potassium, and their corresponding control rates. The secondary outcome was the change in RAAS activity.

### Management of blood pressure and serum potassium

The patients were prepared from the pharmacological standpoint by stopping spironolactone and other diuretics at least 4 weeks and ACEI and ARB medications at least 2 weeks before SIT; all patients were put on a long-acting Ca^2+^ channel blocker at the time when these measurements were made. From the first day of the trial, patients were given nifedipine controlled-release tablets (30 mg/d) to lower BP. During the run-in period, the drug was discontinued if the subject’s BP was less than 90/60mmHg, and drug dosage increased to 60 mg/d if the subject had an increase in BP (>180/110 mmHg). During the entire dietary intervention, the BP-lowering protocol was the same as in the run-in period. There was no potassium supplementation during the trial, and patients were withdrawn from the study if they could not tolerate the diet or if their serum potassium fell below 2.8 mmol/L.

### Diet management

Meal recipes were designed by professional nutritionists in our hospital. Based on dietary guidelines of China (37) and studies of low-sodium diets in essential hypertension (27), we specified 100 mmol and 50 mmol sodium per day for the normal sodium diet and the low-sodium diet, respectively, and controlled the dietary sodium intake of the participants by adding salt packets (NaCl) to salt-free meals. A daily salt-free meal with 1800 kcal or 1600 kcal was provided for males and females, respectively. The ratio of carbohydrates/protein/fat was 4:3:3. The normal sodium group received 100 mmol sodium per day in both the run-in and the intervention periods, and the low sodium group received 100 mmol sodium per day in the run-in period and 50 mmol sodium per day during the intervention period. The participants consumed all food and salt provided, and were not offered additional food or drink throughout the study.

### Clinical and biochemical tests

Systolic blood pressure (SBP), diastolic blood pressure (DBP), and mean arterial pressure (MAP) = DBP + 1/3 (SBP-DBP) were determined twice a day (7 a.m. and 16 p.m.) using an Omron electronic blood pressure monitor. Patients sat quietly in a quiet environment for 5–10 min before starting BP measurements, and were not allowed to drink strong tea, coffee, or alcohol for the preceding 30 min. The subject’s arm was placed at the same level as the right atrium and abducted to 45°, and the cuff was emptied of gas using a uniform electronic sphygmomanometer with the lower edge of the cuff 2–3 cm from the elbow fossa. BP was measured again at 1–2 min intervals and values averaged. We considered the average of BP at 7 a.m. and 16 p.m.

Peripheral venous blood was collected on the mornings of Day 1 (before the run-in period), Day 8 (end of the run-in period), and Day 15 (end of the intervention period) for routine hematology, liver and kidney function, PRA, PAC, c-reactive protein (CRP), brain natriuretic peptide (BNP), and troponin I (TNI) measurements. We tested serum electrolytes and 24-h urine electrolytes daily, and recorded daily water intake and fasting weight in the morning. Blood samples were kept at -4°C immediately after collection and sent for testing within 2 h.

### Biochemical measurements

Blood samples for clinical chemistry analysis were collected after an overnight fast of at least 10 h. All tests were performed in a College of American Pathologists-accredited laboratory (No. 7217913). Serum aldosterone and plasma renin activities were measured with a radioimmunoassay kit (Beckman Coulter Corp.) following the manufacturer’s instructions. Serum cortisol and serum ACTH were measured *via* immunoluminescence and radioimmunoassay, respectively (Beckman Coulter Corp.), following the manufacturer’s instructions.

### Statistical analysis

SPSS 26.0 software was used for statistical analyses. Normally distributed data were presented as means ± standard deviation (SD) and non-normally distributed data were expressed as median and interquartile intervals (IQ 25-75%). Categorical variables were presented as frequencies or percentages. The *t*-test and chi-square test were used for comparisons between two groups for continuous and categorical variables, respectively. Multivariable regression analysis was performed to investigate the factors influencing BP after dietary interventions. Scatter plots and bar charts were plotted using GraphPad (8.0) software; p < 0.05 on two-side tests was defined as significant. Sample size was calculated based on previous studies, assuming that SBP drops by 4.6 (3.2–5.9) mmHg for patients in the low sodium group and by 2.1 (0.8–3.4) mmHg for patients in the normal sodium group (27). A sample size of 21 patients per group was estimated to provide the trial with 90% power to detect differences at a two-sided alpha level of 0.05.

## Results

### Baseline characteristics of participants

**Figure 2:**
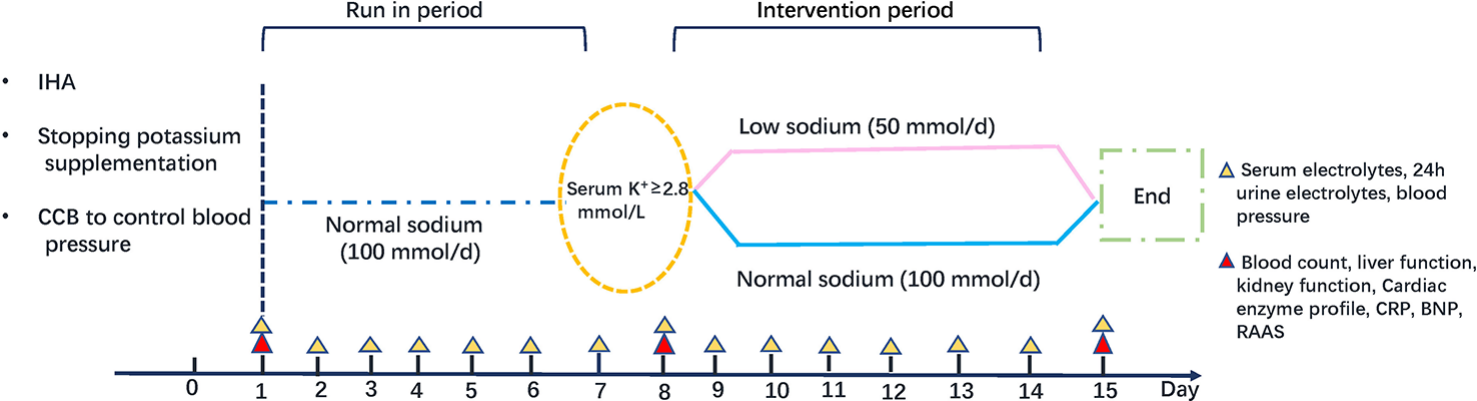
Study timeline.

We recruited 50 IHA patients for the dietary intervention trial and assigned them to the low sodium group or the normal sodium group (25 patients per group) in a randomized manner. The trial’s timeline is shown in **Figure 2**. Baseline characteristics of the two groups are presented in **Table 1**. Before the trial, there was no difference between the normal and low sodium groups in gender (male, 60.0% *vs*. 48.0%, p > 0.05), age (52.2 ± 10.3 *vs*. 53.2 ± 10.7 years, p > 0.05), BMI (26.0 ± 5.1 *vs*. 25.7 ± 4.6 kg/m^2^, p > 0.05), SBP (141.7 ± 12.6 *vs*. 144.0 ± 12.0 mmHg, p > 0.05), DBP (90.2 ± 9.9 *vs*. 91.2 ± 8.7 mmHg, p > 0.05), PRA [0.41 (0.24, 0.83) *vs*. 0.49 (0.34, 0.70) ng/ml/h, p > 0.05], PAC [28.6 (17.9, 45.8) *vs*. 33.0 (21.2, 39.1) ng/dl, p > 0.05], serum sodium (143.4 ± 2.1 *vs*. 142.6 ± 2.0 mmol/L, p > 0.05), and serum potassium (3.23 ± 0.36 *vs*. 3.28 ± 0.29 mmol/L, p > 0.05). Before the trial, the defined daily dose (DDD) (38), i.e. the assumed average maintenance daily dose for a drug used in adults for its main indication, was only mildly different (1.7 ± 1.1 *vs*. 2.0 ± 1.3, p > 0.05) between the two groups.

**Table 1:** Baseline characteristics of participants in the two groups.

	Normal sodium (n = 25)	Low sodium (n = 25)	p
Male (*N*, %)	12 (48.0)	15 (60.0)	0.395
Age (y)	53.2 ± 10.7	52.2 ± 10.3	0.758
BMI (kg/m^2^)	25.7 ± 4.6	26.0 ± 5.1	0.818
DDD	1.7 ± 1.1	2.0 ± 1.3	0.396
Potassium chloride supplementation (g/d)	2.9 ± 2.7	2.7 ± 2.3	0.730
SBP (mmHg)	144.0 ± 12.0	141.7 ± 12.6	0.525
DBP (mmHg)	91.2 ± 8.7	90.2 ± 9.9	0.698
MAP (mmHg)	108.8 ± 8.6	107.4 ± 10.0	0.590
Duration of HT (y)	10.0 (4.5, 14.5)	10.0 (1.6, 17.5)	0.861
Cortisol (µg/dl)	12.0 (9.3, 16.2)	11.2 (9.7, 15.4)	0.786
ACTH (pg/ml)	36.0 (23.6, 44.6)	27.8 (23.6, 34.8)	0.265
PRA (ng/ml/h)	0.49 (0.34, 0.70)	0.41 (0.24, 0.83)	0.655
PAC (ng/dl)	33.0 (21.2, 39.1)	28.6 (17.9, 45.8)	0.541
ARR [(ng/dl)/(ng/ml/h)]	70.9 (35.2, 111.6)	51.2 (34.6, 124.4)	0.793
Serum Na^+^ (mmol/L)	142.6 ± 2.0	143.4 ± 2.1	0.205
Serum K^+^ (mmol/L)	3.28 ± 0.29	3.23 ± 0.36	0.575
Ccr (ml/min)	85.3 ± 23.1	91.7 ± 22.6	0.328
Uric acid (µmol/L)	326.3 ± 85.1	315.2 ± 96.0	0.144

BMI, body mass index; DDD, defined daily dose; SBP, systolic blood pressure; DBP, diastolic blood pressure; MAP, mean arterial pressure; HT, hypertension; ACTH, adrenal corticotropic hormone; PRA, plasma renin activity; PAC, plasma aldosterone concentration; ARR, aldosterone to renin ratio; Na^+^, sodium; K^+^, potassium; Ccr, creatinine clearance rate.

Normally distributed data are presented as means ± standard deviations and non-normally distributed data are expressed as median and interquartile intervals (IQ 25%–75%). Categorical variables are presented as percentages.

### Changes in clinical and biochemical parameters after the dietary intervention

**Figure 3:**
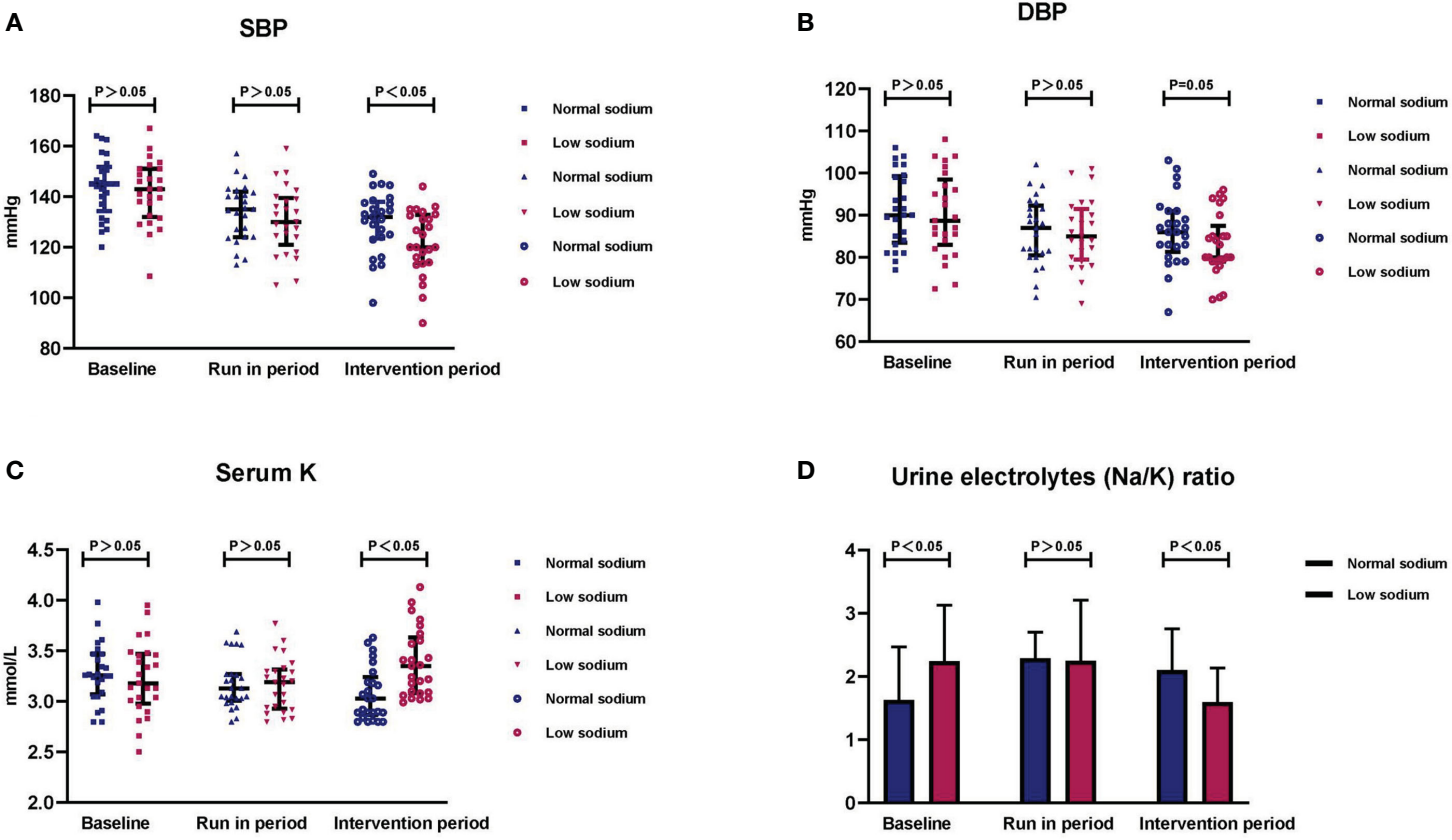
Changes in blood pressure, serum potassium, and urinary electrolytes after the dietary intervention. Summary data of **(A)** SBP, **(B)** DBP, **(C)** serum potassium, and **(D)** 24-h urine sodium to potassium ratio (Na^+^/K^+^) in the two study groups after the intervention period. Data are shown as median with interquartile ranges.

During the run-in period two participants (one in the low sodium group and the other in the normal sodium group) had BP <90/60 mmHg and discontinued their antihypertensive medication. Another participant in the normal sodium diet group had BP consistently greater than 180/110 mmHg and his antihypertensive medication was increased to 60 mg/d. Parameters after the dietary intervention for both groups are presented in **Table 2**. After the 14-day dietary intervention, there was a slight, non-significant decrease in body weight in both groups. After the run-in period, BP decreased comparably in the normal and low sodium groups: SBP, 134.0 ± 11.3 *vs*. 130.7 ± 13.4 mmHg, p > 0.05; DBP, 86.2 ± 8.0 *vs*. 85.8 ± 8.1 mmHg, p > 0.05; MAP, 102.1 ± 7.8 *vs*.100.8 ± 8.8 mmHg, p > 0.05, respectively. At the end of the intervention period, no significant change in BP was detected in patients in the normal sodium group, whereas a further decrease in BP was recorded in those in the low sodium group. Respective BP values were: SBP, 129.9 ± 12.1 *vs*. 121.8 ± 12.8 mmHg, p < 0.05 (**Figure 3A**); DBP, 86.4 ± 8.2 *vs*. 82.6 ± 7.6 mmHg, p < 0.05 (**Figure 3B**); and MAP, 100.9 ± 8.4 *vs*. 95.7 ± 8.8 mmHg, p < 0.05.

**Table 2:** Results of the dietary interventions.

	Normal sodium (n = 25)		Low sodium (n = 25)			
	Run-in period	Intervention period	Run-in period	Intervention period	p*^I^*	p*^II^*
Weight (kg)	67.8 ± 13.3	67.0 ± 13.0	72.0 ± 16.3	71.7 ± 16.1	0.543	0.216
SBP (mmHg)	134.0 ± 11.3	129.9 ± 12.1	130.7 ± 13.4	121.8 ± 12.8	0.508	0.005
DBP (mmHg)	86.2 ± 8.0	86.4 ± 8.2	85.8 ± 8.1	82.6 ± 7.6	0.936	0.039
MAP (mmHg)	102.1 ± 7.8	100.9 ± 8.4	100.8 ± 8.8	95.7 ± 8.8	0.759	0.010
Heart rate (N/min)	71.1 ± 8.6	73.8 ± 10.6	72.2 ± 7.6	73.9 ± 10.2	0.636	0.869
Serum Na^+^ (mmol/L)	143.2 ± 1.7	143.5 ± 1.8	143.3 ± 1.9	142.8 ± 2.0	0.908	0.100
Serum K^+^ (mmol/L)	3.18 ± 0.25	3.07 ± 0.27	3.17 ± 0.26	3.38 ± 0.33	0.910	<0.001
24-h urine Na^+^ (mmol/L/24 h)	97.8 ± 16.7	96.1 ± 27.0	93.9 ± 16.6	54.3 ± 24.5	0.278	<0.001
24-h urine K^+^ (mmol/L/24 h)	48.0 ± 15.0	45.4 ± 13.9	42.1 ± 15.5	33.0 ± 10.8	0.054	0.003
PRA (ng/ml/h)	0.60 (0.41, 1.28)	0.78 (0.49, 1.45)	0.43 (0.29, 0.96)	0.63 (0.33, 1.03)	0.110	0.363
PAC (ng/dl)	28.2 (18.9, 40.7)	29.9 (20.5, 35.5)	21.7 (19.1, 34.5)	26.6 (20.7, 54.1)	0.479	0.992
ARR [(ng/dl)/(ng/ml/h)]	47.4 (19.2, 81.2)	40.3 (21.4, 65.4)	53.7 (32.9, 110.8)	38.6 (30.2, 81.7)	0.412	0.674
Creatinine (µmol/L)	77.8 ± 20.3	78.7 ± 20.8	81.7 ± 20.2	81.9 ± 22.3	0.284	0.807
Ccr (ml/min)	87.1 ± 22.2	85.3 ± 21.5	90.6 ± 20.3	92.1 ± 18.9	0.948	0.456
Uric acid (µmol/L)	331.7 ± 89.5	315.2 ± 96.0	303.8 ± 92.1	318.0 ± 103.0	0.774	0.004
BNP (pg/ml)	53.7 (29.4, 79.4)	61.1 (33.4, 83.3)	38.7 (20.9, 80.0)	32.3 (20.0, 74.9)	0.382	0.123
CRP (mg/L)	1.5 (0.9, 2.0)	1.2 (1.0, 2.7)	1.0 (0.7, 2.0)	0.8 (0.5, 1.1)	0.716	0.042
Urine electrolytes (Na^+^/K^+^) ratio	2.3 (1.6, 2.8)	2.1 (1.6, 2.8)	2.3 (1.7, 3.2)	1.6 (1.2, 2.1)	0.503	0.041
DDD	1.0 ± 0.3	1.0 ± 0.3	1.0 ± 0.3	1.0 ± 0.3	1.000	1.000

SBP, systolic blood pressure; DBP, diastolic blood pressure; MAP, mean arterial pressure; Na^+^, sodium; K^+^, potassium; PRA, plasma renin activity; PAC, plasma aldosterone concentration; ARR, aldosterone to renin rate; Ccr, creatinine clearance rate; HCT, hematocrit test; LDH, lactate dehydrogenase; CK-MB, creatine kinase isoenzyme-MB; BNP, brain natriuretic peptide; CRP, c-reactive protein.

^I^ Comparison between the two groups after the run-in period.

^II^ Comparison between the two groups after the intervention period.

t-Test results from the run-in period were corrected for baseline data, and t-test results from the intervention period were corrected for run-in period data.

After discontinuation of potassium supplementation, serum potassium decreased similarly in both groups after the run-in period (3.18 ± 0.25 *vs*. 3.17 ± 0.26 mmol/L for the normal and low sodium groups, respectively; p > 0.05). After the intervention period, serum potassium did not change significantly in the normal sodium group, and was significantly increased in the low sodium group (3.07 ± 0.27 *vs*. 3.38 ± 0.33 mmol/L, respectively; p < 0.001) (**Figure 3C**). Serum sodium remained relatively stable during the trial in both groups.

**Supplementary Table 1** shows the changes of parameters (SBP, DBP, MAP, and serum potassium) within the low and normal sodium groups. Patients who received 100 mmol/d sodium (normal sodium diet) during the run-in period had significant reductions in BP (SBP mean decrease of 10.1 ± 11.0 mmHg, DBP mean decrease of 5.0 ± 7.0 mmHg, and MAP mean decrease of 6.7 ± 7.4 mmHg), and the above indicators did not change significantly after the intervention period. Similarly, patients in the low sodium group had a significant decrease in BP after the run-in period (SBP mean decrease 11.1 ± 11.0 mmHg, DBP mean decrease 4.4 ± 8.2 mmHg, MAP, mean decrease 6.6 ± 8.5 mmHg), and a further decrease after the intervention period (SBP mean decrease 8.8 ± 6.1 mmHg, DBP mean decrease of 3.2 ± 6.3 mmHg, MAP mean decrease of 5.1 ± 5.2 mmHg). After the intervention period, the mean increase in potassium was 0.22 ± 0.20 mmol/L in the low sodium group, and it was not increased in the normal sodium group.

After the run-in period, no significant differences between the low and normal sodium groups were detected for 24-h urinary sodium (93.9 ± 16.6 *vs*. 97.8 ± 16.7 mmol/L/24 h, p > 0.05), 24-h urinary potassium (42.1 ± 15.5 *vs*. 48.0 ± 15.0 mmol/L/24 h, p > 0.05), and urinary electrolyte ratio (sodium/potassium) [2.3 (1.7, 3.2 *vs*. 2.3 (1.6, 2.8), p > 0.05]. After the intervention period, these parameters were all significantly lower in the low sodium group compared to the normal sodium group [24-h urinary sodium, 54.3 ± 24.5 *vs*. 96.1 ± 27.0 mmol/L/24 h, p < 0.05; 24-h urinary potassium, 33.0 ± 10.8 *vs*. 45.4 ± 13.9 mmol/L/24 h, p < 0.05; urinary electrolyte ratio, 1.6 (1.2, 2.1) *vs*. 2.1 (1.6, 2.8), p < 0.05 (**Figure 3D**)].

After the run-in period, the low and normal sodium groups showed no differences in PRA [0.60 (0.41, 1.28) *vs*. 0.43 (0.29, 0.96) ng/ml/h, p > 0.05] or PAC [28.2 (18.9, 40.7) *vs*. 21.7 (19.1, 34.5) ng/dl, p > 0.05]. After the intervention phase, both PRA and PAC were mildly increased in the low and normal sodium groups, with no significant intergroup differences: PRA [0.78 (0.49, 1.45) *vs*. 0.63 (0.33, 1.03) ng/ml/h, p > 0.05], PAC [29.9 (20.5, 35.5) *vs*. 26.6 (20.7, 54.1) ng/dl, p > 0.05]. After the intervention period, CRP was lower in the low sodium group than in the normal sodium group [0.8 (0.5, 1.1) *vs*. 1.2 (1.0, 2.7) mg/L, respectively; p < 0.05]. In turn, BNP was significantly decreased in both groups, attaining post-trial values that were not statistically different from each other [32.3 (20.0, 74.9) *vs*. 61.1 (33.4, 83.3) pg/ml, p > 0.05]. After the dietary intervention, uric acid decreased in patients in the normal sodium group (from 331.7 ± 89.5 to 315.2 ± 96.0 µmol/L) and increased in patients in the low sodium group (from 303.8 ± 92.1 to 318.0 ± 103.0 µmol/L), with a statistical difference noted between post-intervention values for the two groups (315.2 ± 96.0 *vs*. 318.0 ± 103.0 µmol/L, p < 0.05). There was no statistical difference in urea nitrogen and Ccr between the two groups.

### Serum potassium and BP control rates

We analyzed the control rates of BP and serum potassium at different levels after the dietary intervention (**Table 3**). Control rates for SBP <120 mmHg (48.0% *vs*. 20.0%, p < 0.05) and DBP <80 mmHg (52.0% *vs*. 20.0%, p < 0.05) were higher in the low sodium group, whereas the normal sodium group exhibited higher control of 80≤ DBP <90 mmHg (52.0% *vs*. 24.0%, p < 0.05). There was no significant difference in the control of other BP levels between the two groups (p > 0.05). In addition, the SBP/DBP control rate in the low sodium group was overall higher than in the normal sodium group: <140/90 mmHg, 76.0% *vs*. 64.0%, respectively; p > 0.05; mean, 70.0%), <130/85 mmHg, 56.0% *vs*. 20.0%; p < 0.05; mean, 38.0%), <120/80 mmHg, 44.0% *vs*. 12.0%; p < 0.05; mean, 28.0%).

**Table 3:** Control rates for serum potassium and blood pressure after the dietary intervention.

	Total (n = 50) N (%)	Normal sodium (n = 25) N (%)	Low sodium (n = 25) N (%)	p
SBP (mmHg)				
140≤SBP<150	5 (10.0)	4 (16.0)	1 (4.0)	0.349
130≤SBP<140	19 (38.0)	11 (44.0)	8 (32.0)	0.382
120≤SBP<130	9 (18.0)	5 (20.0)	4 (16.0)	1.000
SBP<120	17 (34.0)	5 (20.0)	12 (48.0)	0.037
DBP (mmHg)				
100≤DBP<110	2 (4.0)	2 (8.0)	0 (0.0)	0.490
90≤DBP<100	11 (22.0)	5 (20.0)	6 (24.0)	0.733
80≤DBP<90	19 (38.0)	13 (52.0)	6 (24.0)	0.041
DBP<80	18 (36.0)	5 (20.0)	13 (52.0)	0.018
SBP/DBP (mmHg)				
<140/90	35 (70.0)	16 (64.0)	19 (76.0)	0.355
<130/85	19 (38.0)	5 (20.0)	14 (56.0)	0.019
<120/80	14 (28.0)	3 (12.0)	11 (44.0)	0.012
Serum K^+^ (mmol/L)				
≥3.5	11 (22.0)	3 (12.0)	8 (32.0)	0.088
3.2≤K^+^<3.5	11 (22.0)	3 (12.0)	8 (32.0)	0.088
3.0≤K^+^<3.2	15 (30.0)	7 (28.0)	8 (32.0)	0.758
2.8≤K^+^<3.0	13 (26.0)	12 (48.0)	1 (4.0)	<0.001

SBP, systolic blood pressure; DBP, diastolic blood pressure; MAP, mean arterial pressure; K^+^, potassium.

After the intervention period, patients in the low sodium group had, compared to the normal sodium group, a higher but not significantly different control rate of serum potassium ≥3.5 mmol/L (32.0% *vs*. 12.0%, p > 0.05), 3.2≤ serum potassium <3.5 mmol/L (32.0% *vs*. 12.0%, p > 0.05), and 3.0≤ serum potassium <3.2 mmol/L (32.0% *vs*. 28.0%, p > 0.05). The proportion of patients with serum potassium <3.0 mmol/L was higher in the normal sodium group (48.0% *vs*. 4.0%, p < 0.05).

### Factors affecting BP control

Based on the 2018 ESC/ESH guidelines (39) and the specificity of PA, we defined BP (SBP/DBP) <120/80 mmHg as BP control and compared the differences between the controlled BP and the uncontrolled BP groups (**Table 4**). After the trial, 14 of the 50 patients (28.0%) had controlled BP. Patients in the controlled BP group had the following characteristics: more females (71.4% *vs*. 36.1%, p < 0.05), lower SBP (135.0 ± 12.6 *vs*. 145.9 ± 10.8 mmHg, p < 0.05), and lower MAP (103.4 ± 10.2 *vs*. 109.9 ± 8.3 mmHg, p < 0.05) at baseline, and were mostly allocated to the low-sodium diet (78.6% *vs*. 38.9%, p < 0.05). There were no differences in baseline DBP, DDD, PRA, PAC, ARR, serum sodium, and serum potassium between groups (all p > 0.05). The univariate logistic regression analysis showed significant associations for female gender [OR (CI 95%): 0.226 (0.059, 0.867), p < 0.05], SBP at baseline [0.918 (0.861, 0.979), p < 0.05], MAP at baseline [0.919 (0.851, 0.919), p < 0.05], and low-sodium diet [0.174 (0.041, 0.734), p < 0.05] and controlled BP (<120/80 mmHg). Further multiple regression analysis revealed that female gender [0.157 (0.026, 0.959), p < 0.05] and low-sodium diet [0.070 (0.009, 0.519), p < 0.05] were independent protective factors for controlled BP (**Table 5**).

**Table 4:** Demographic and clinical characteristics of patients with controlled and uncontrolled BP after the dietary intervention.

	Uncontrolled BP (≥120/80 mmHg)	Controlled BP (<120/80 mmHg)	p
Patients, *N* (%)	36 (72.0)	14 (28.0)	
Age (y)	53.9 ± 11.2	49.6 ± 7.4	0.127
Female, *N* (%)	13 (36.1)	10 (71.4)	0.024
BMI^1^ (kg/m^2^)	26.3 ± 4.5	24.6 ± 5.6	0.253
DM, *N* (%)	10 (27.8)	4 (28.6)	1.000
Duration of HT (y)	10.0 (4.0, 19.5)	7.0 (1.6, 12.8)	0.204
SBP^1^ (mmHg)	145.9 ± 10.8	135.0 ± 12.6	0.003
DBP^1^ (mmHg)	91.9 ± 8.8	87.6 ± 10.0	0.138
MAP^1^ (mmHg)	109.9 ± 8.3	103.4 ± 10.2	0.023
PRA^1^ (mg/dl/h)	0.48 (0.33, 0.73)	0.41 (0.23, 0.79)	0.604
PAC^1^ (ng/dl)	29.4 (17.9, 44.0)	29.9 (25.4, 42.0)	0.517
ARR^1^	58.2 (31.1, 111.0)	56.2 (42.1, 143.7)	0.450
Serum K^+^ (mmol/L)	3.21 ± 0.26	3.37 ± 0.44	0.211
Serum Na^+^ (mmol/L)	143.2 ± 2.0	142.4 ± 2.4	0.252
24-h urine Na^+^ (mmol/L/24 h)	93.0 (62.9, 117.3)	101.2 (83.6, 125.2)	0.359
24-h urine K^+^ (mmol/L/24 h)	48.0 (37.0, 57.4)	53.0 (26.3, 65.3)	0.754
Low-sodium diet, *N* (%)	14 (38.9)	11 (78.6)	0.012
Serum K^+^≥3.5^2^, *N* (%)	6 (16.7)	5 (35.7)	0.252
DDD^1^	2.0 ± 1.1	1.7 ± 1.4	0.542

BMI, body mass index; DM, diabetes mellitus; HT, hypertension; SBP, systolic blood pressure; DBP, diastolic blood pressure; MAP, mean arterial pressure; PRA, plasma renin activity; PAC, plasma aldosterone concentration; ARR, aldosterone to renin rate; Na^+^, sodium; K^+^, potassium; DDD, defined daily dose.

^1^, before the run-in period, ^2^, after the intervention period.

**Table 5:** Multiple regression analysis of factors influencing BP after the dietary intervention.

	Univariate		Multivariate	p
	OR (CI 95%)	*P*	OR (CI 95%)	
Age (y)	0.960 (0.903, 1.021)	0.196		
Female, *N* (%)	0.226 (0.059, 0.867)	0.030	0.157 (0.026, 0.959)	0.045
Duration of HT (y)	0.940 (0.860, 1.026)	0.167		
SBP^1^ (mmHg)	0.918 (0.861, 0.979)	0.009	0.861 (0.726, 1.022)	0.087
DBP^1^ (mmHg)	0.947 (0.881, 1.018)	0.141		
MAP^1^ (mmHg)	0.919 (0.851, 0.919)	0.032	1.092 (0.906, 1.316)	0.355
PRA^1^ (ng/ml/h)	1.062 (0.427, 2.640)	0.897		
PAC^1^ (ng/dl)	1.005 (0.966, 1.046)	0.799		
ARR^1^ [(ng/dl)/(ng/ml/h)]	1.005 (0.995, 1.015)	0.320		
Low-sodium diet, *N* (%)	0.174 (0.041, 0.734)	0.017	0.070 (0.009, 0.519)	0.009

HT, hypertension; SBP, systolic blood pressure; DBP, diastolic blood pressure; MAP, mean arterial pressure; PRA, plasma renin activity; PAC, plasma aldosterone concentration; ARR, aldosterone to renin rate.

^1^, before the run-in period.

## Discussion

Recent studies reported high prevalence of PA (29.1%) in patients with resistant hypertension (40) and association of this condition with severe target organ damage and cardiovascular events (41). IHA accounts for 60% of PA cases (9), is defined by excess aldosterone originating from bilateral adrenal glands, and is usually treated medically with mineralocorticoid receptor antagonists (MRAs) (42). These regimens have the disadvantage of being poorly tolerated when used at high doses. Therefore, we conducted this study with the aim of evaluating a non-pharmacological alternative to MRA or measures aimed at helping reduce its dosage. Our study has several important findings. First, it suggests a specific recommended sodium intake, which could be an important addition to current guidelines (43), and highlights that sodium levels in food ingredients should not be ignored. After the run-in period (100 mmol/d sodium), BP decreased more in both groups (SBP ~10 mmHg, MAP ~6.6 mmHg) and there was a further decrease in BP (SBP ~8.8 mmHg, MAP ~5.1 mmHg) after the intervention period in patients in the low sodium group (50 mmol/d sodium). Similar results were reported in the Dietary Approaches to Stop Hypertension (DASH) study (27), although the latter was conducted in patients with mild hypertension. There are a number of factors that can influence the effect of dietary sodium on BP. For instance, a study showed that the antihypertensive effect of a low-sodium diet varies with ethnicity and BP levels (22), but our study does not have these caveats.

Second, in our study the total control rate of SBP <120 mmHg was 34.0% (17/50 patients), with greater representation of patients in the low, compared to the normal, sodium group (48.0% *vs*. 20.0%, respectively; p < 0.05). Meanwhile, the total control rate of DBP <80 mmHg was 36.0% (18/50 patients) with, again, a higher control rate recorded in the low sodium group (52.0% *vs*. 20.0% in the normal sodium group; p < 0.05). This implies that reducing dietary sodium intake can lead to better control of BP in IHA. We also found that a low-sodium diet provides better control of BP (SBP/DBP <120/80 mmHg) in female patients. It is well known that sex hormones play an important role in BP regulation. Premenopausal women typically have a lower BP compared to age-matched men, and both endogenous and exogenous estrogens have hypotensive effects (44–46). Therefore, in line with mounting evidence that a low-sodium diet complements other non-pharmacological and pharmacological interventions aimed at lowering BP, we recommend a low-sodium diet as a first consideration for managing BP in patients, and specially women, with IHA. Third, our data showed that reducing sodium intake in IHA can effectively raise serum potassium levels. Normal kidney function is essential for regulating the balance of sodium and potassium in the body, as renal potassium excretion is influenced by sodium intake (47). When sodium intake decreases, urinary potassium elimination is reduced, and serum potassium increases. Even after discontinuing of potassium supplementation, before initiating the trial, serum potassium decreased only mildly (mean reduction of 0.08 ± 0.33 mmol/L) after supplying 100 mmol sodium intake per day during the run-in period. Moreover, after the trial, serum potassium levels were augmented (mean increase of 0.22 ± 0.20 mmol/L) in the low sodium group. Although serum potassium exceeded 3 mmol/L in both groups after reducing sodium intake, the average level was significantly higher in the low, compared to the normal, sodium diet group (3.38 ± 0.33 *vs*. 3.07 ± 0.27 mmol/L, respectively; p < 0.001), which may favor reducing or eliminating the need for potassium supplements or MRAs. Worthy of note, we found that by the end of the trial (conducted without potassium supplementation), in the low sodium group 96.0% of patients had serum potassium ≥3.0 mmol/L, 64.0% had serum potassium ≥3.2 mmol/L, and 32.0% had serum potassium ≥3.5 mmol/L. Therefore, serum potassium levels can be effectively raised by reducing sodium intake.

Changes in PRA following reduced sodium intake may be beneficial for IHA. Hundemer et al. (35) showed that PRA may be a predictor of cardiovascular outcomes and may serve as an important indicator of treatment response. In our study, PRA rose in both groups after the dietary intervention, but there was no difference between the two groups. This may be due to the short duration of the trial and/or inadequate sample size. Excessive sodium intake inhibits the RAAS, which in turn reduces sodium reabsorption and thus promotes its excretion (48). Furthermore, IHA patients have an elevated NaCl taste threshold, influenced by high aldosterone levels (17), which determines a daily sodium intake significantly higher than that specified by dietary guideline recommendations (18). Therefore, a low-sodium diet is fundamentally apt to control BP and serum potassium and to improve long-term prognosis in IHA patients.

In conclusion, reducing dietary sodium was effective in lowering BP and raising serum potassium in patients with IHA who were maintained on low-dose antihypertensive medications without potassium supplementation. These effects were more pronounced in participants consuming 50 mmol, rather than 100 mmol, sodium per day over the trial’s duration. Specifically, in the low-sodium group (50 mmol/d sodium) 44.0% of patients had normal BP (<120/80 mmHg) and 32.0% of patients had normal serum potassium (3.5–5.5 mmol/L) at the end of the trial. In addition, the low-sodium diet was associated with better BP control in female IHA patients.

There are some limitations in our study. First, as it lasted only 2 weeks, potential long-term effects of the low-sodium diet on IHA need to be further explored. Second, excessive aldosterone concentrations persist after adopting a low-sodium diet, an issue that may be adequately addressed by low-dose MRA treatment.

## Data availability statement

The data analyzed in this study is subject to the following licenses/restrictions: Data available on request from the authors. Requests to access these datasets should be directed to Lihua Zhou, zlh1880123@126.com.

## Ethics statement

The studies involving human participants were reviewed and approved by Ruijin Hospital Ethics Committee Shanghal JiaoTong University School of Medicine. The patients/participants provided their written informed consent to participate in this study.

## Author contributions

LZ, YJ and WW contributed to conception and design of the study. LZ organized the database and performed the statistical analysis. LZ and YJ wrote the first draft of the manuscript. CZ, TS, LJ, WZ, XZ, and LW wrote sections of the manuscript. Both LZ and YJ are responsible for research design and implementation, data analysis, and writing the manuscript. All authors contributed to manuscript revision, read, and approved the submitted version.

## Supplementary material

Supplementary Table 1 Intra-group comparison of blood pressure and serum potassium. Normal-G, normal sodium diet group; Low-G, low-sodium diet group; SBP, systolic blood pressure; DBP, diastolic blood pressure; MAP, mean blood pressure; K^+^, potassium; BP-related variables expressed in mmHg; Serum K^+^ levels expressed in mmol/L. p^0^, comparison of baseline and run-in periods; p^1^, comparison of run-in and intervention periods; p^2^, comparison of baseline and intervention periods; 1 #, change between baseline and run-in period; 2 #, change between run-in and intervention period.
