# Supplementary material for: Effects of a low-sodium diet in patients with idiopathic hyperaldosteronism: a randomized controlled trial
Source: Front Endocrinol (Lausanne). 2023 Apr 19;14:1124479. doi: 10.3389/fendo.2023.1124479 (PMC10154588; doi:10.3389/fendo.2023.1124479)
Supplement: Supplementary Table 1 — Intra-group comparison of blood pressure and serum potassium. Normal-G, normal sodium diet group; Low-G, low-sodium diet group; SBP, systolic blood pressure; DBP, diastolic blood pressure; MAP, mean blood pressure; K+, potassium; BP-related variables expressed in mmHg; Serum K+ levels expressed in mmol/L. p0, comparison of baseline and run-in periods; p1, comparison of run-in and intervention periods; p2, comparison of baseline and intervention periods; 1 #, change between baseline and run-in period; 2 #, change between run-in and intervention period. [file Table_1.docx]

**Supplementary Table 1**

**Table S1** Intra-group comparison of blood pressure and serum potassium.

| **Groups** | **SBP-baseline** | **SBP-run-in period** | **SBP-intervention period** | **△SBP1** **#** | **△SBP2** **#** | ***P*^0^** | ***P*^1^** | ***P*^2^** |
| --- | --- | --- | --- | --- | --- | --- | --- | --- |
| Normal-G | 144.0±12.0 | 134.0±11.3 | 129.9±12.1 | 10.1±11.0 | 4.0±6.6 | 0.004 | 0.234 | ＜0.001 |
| Low-G | 141.7±12.6 | 130.7±13.4 | 121.8±12.8 | 11.1±11.0 | 8.8±6.1 | 0.004 | 0.021 | ＜0.001 |
|  |  |  |  |  |  |  |  |  |
| **Groups** | **DBP-baseline** | **DBP-run-in period** | **DBP-intervention period** | **△DBP1** | **△DBP2** | ***P*^0^** | ***P*^1^** | ***P*^2^** |
| Normal-G | 91.2±8.7 | 86.2±8.0 | 86.4±8.2 | 5.0±7.0 | -0.2±6.3 | 0.040 | 0.938 | 0.050 |
| Low-G | 90.2±9.9 | 85.8±8.1 | 82.6±7.6 | 4.4±8.2 | 3.2±6.3 | 0.094 | 0.158 | 0.004 |
|  |  |  |  |  |  |  |  |  |
| **Groups** | **MAP-baseline** | **MAP-run-in period** | **MAP-intervention period** | **△MAP1** | **△MAP2** | ***P*^0^** | ***P*^1^** | ***P*^2^** |
| Normal-G | 108.8±8.6 | 102.1±7.8 | 100.9±8.4 | 6.7±7.4 | 1.2±5.8 | 0.006 | 0.598 | 0.002 |
| Low-G | 107.4±10.0 | 100.8±8.8 | 95.7±8.8 | 6.6±8.5 | 5.1±5.2 | 0.017 | 0.047 | ＜0.001 |
|  |  |  |  |  |  |  |  |  |
| **Groups** | **K^+^-baseline** | **K^+^-run-in period** | **K^+^-intervention period** | **△K^+^1** | **△K^+^ 2** | ***P*^0^** | ***P*^1^** | ***P*^2^** |
| Normal-G | 3.28±0.29 | 3.18±0.25 | 3.07±0.27 | 0.11±0.37 | 0.10±0.23 | 0.171 | 0.169 | 0.011 |
| Low-G | 3.23±0.36 | 3.17±0.26 | 3.38±0.33 | 0.06±0.28 | -0.22±0.20 | 0.494 | 0.014 | 0.122 |

Normal-G, normal sodium diet group; Low-G, low-sodium diet group; SBP, systolic blood pressure; DBP, diastolic blood pressure; MAP, mean blood pressure; K^+^, potassium; BP-related variables expressed in mmHg; Serum K^+^ levels expressed in mmol/L.

*P*^0^, comparison of baseline and run-in periods; *P*^1^, comparison of run-in and intervention periods; *P*^2^, comparison of baseline and intervention periods; 1 #, change between baseline and run-in period; 2 #, change between run-in and intervention period.
